# Applications of novel vaccines to immotherapy of food allergy

**DOI:** 10.1186/2045-7022-1-S1-S79

**Published:** 2011-08-12

**Authors:** Mübeccel Akdis

**Affiliations:** 1 Swiss Institute for Allergy and Asthma Research (SIAF), Davos, Switzerland

## 

Allergic diseases including atopic dermatitis (AD), food allergy, allergic rhinitis, asthma and anaphylaxis have significant associated morbidity along with large health-care expenditures. Food allergies are caused by immune responses to food proteins and represent a breakdown of oral tolerance. They can range from mild pruritus to life-threatening anaphylaxis. The only treatment is food avoidance, which might cause near-fatal and fatal reactions by accidental exposures. For this reason, there has been recent interest in immunotherapy, which may induce desensitization and even tolerance. Through these effects, immunotherapy may decrease the potential for adverse serious reactions with accidental ingestions. Therefore, it is expected that immunotherapy may be adopted as the first treatment to modify the natural history of food allergy.

Allergen-specific immunotherapy (SIT) has been used as a desensitizing therapy for allergic diseases and may represents a curative and specific way of the treatment. However, current allergen-SIT has several disadvantages related to the content of the vaccine, type of adjuvant, route of application, long duration time, side effects, and sometimes limited efficacy. Immune system behaves in a different way to extracellular pathogens as bacteria and parasites. Initially capture of exogenous pathogen by dendritic cell results in phagocytosis, which is then followed by migration to local lymph nodes through chemotactic signals where DCs maturate and lose phagocytic capacity and improve antigen presentation capacity to T cells. In T cell activation, several signals are essential for the differentiation of naive T cells to cytokine-producing effector Th cells. After the discovery of regulatory T cells the concepts of immune regulation have substantially changed during the last decade. Peripheral T-cell tolerance is a key immunologic mechanism in healthy immune response to self-antigens and non-infectious non–self-antigens. Both naturally occurring CD4+CD25+ regulatory T (Treg) cells and inducible populations of allergen-specific, IL-10-secreting Treg type 1 cells contribute to the control of allergen-specific immune responses in several ways: suppression of antigen-presenting cells that support the generation of effector Th2 and Th1 cells; suppression of Th2 and Th1 cells; suppression of mast cells, basophils and eosinophils; interaction with resident tissue cells and remodeling. In addition to the above mechanisms, Tr1 and CD4+CD25+ Treg cells suppress IgE and induce the noninflammatory antibody isotype IgG4.

As a novel approach, direct vaccine administration into lymph nodes and targeting the MHC class II antigen presentation pathway has been hypothesized to increase the immunogenicity, efficacy and the safety of immunotherapy because of low allergen dose, however better presented to T cells. The major cat dander allergen Fel d 1 was fused to a TAT-derived protein translocation domain and to a truncated invariant chain (MAT-Fel d 1). This MAT-Fel d 1 vaccine is efficiently internalized by APCs and induces IL-10 and IFN-g dominated response, but low Th2 cytokines' production in PBMCs of allergic individuals. In a double-blind placebo-controlled clinical trial, MAT-Fel d 1 vaccine adsorbed to alum was administered by 3 intralymphatic injections in increasing dose (1 µg, 3 µg, 10 µg) into inguinal, subcutaneous lymph node within 2 months with 4 weeks intervals. Cat allergic patients became tolerant to nasal challenge with cat dander after only 3 injections. The blood for cell cultures have been taken before the therapy and twice after finishing the treatment: one week and one year respectively. Fel d 1-specific T cell tolerance was observed in the MAT-Fel d 1 group compared to placebo group after one year and the significant enhancement of IL-10 production measured in supernatants correlated with the rise of specific IgG4 in plasma samples. In addition, we observed tendency of increase in Fel d 1-specific CD3+CD4+FOXP3+ T cells’ number in MAT-Fel d 1 treated patients using MHC class II peptide tetramers. Specific IgE production however rose during ILIT but it was contrary to the lack of drug related side effects. These data demonstrate that intralymphnode administration of MAT-Fel d 1 induces allergen-specific immune tolerance in cat allergic patients.
